# Demystifying HIV: academic training for undergraduate nursing students

**DOI:** 10.1590/1980-220X-REEUSP-2026-0023en

**Published:** 2026-09-07

**Authors:** Naomi Tamima Estevam Cipriano, Jacks Soratto, Cristiane Damiani Tomasi

**Affiliations:** 1 Universidade Federal de Santa Catarina, Florianópolis, SC, Brazil.; 2 Universidade do Extremo Sul Catarinense, Criciúma, SC, Brazil.

**Keywords:** HIV, Nursing Care, Education, Nursing, Problem-Based Learning

## Abstract

**Objective:**

To develop and evaluate a participatory and dialogic health education program with nursing students at a community college in Criciúma, SC, on the Human Immunodeficiency Virus and its care pathway.

**Method:**

Action research, grounded in Critical Pedagogy, conducted with 19 nursing students in their 8th through 10th semesters of the program. The training took place in four stages: exploratory, planning, action, and evaluation, with questionnaires administered before and after the intervention.

**Results:**

Before the training, self-assessments of knowledge regarding Pre-Exposure Prophylaxis, Post-Exposure Prophylaxis, and Antiretroviral Therapy were predominantly rated as fair or poor/very poor. After the intervention, all participants rated their knowledge as very good or good. There was also an increase in the perceived readiness to welcome and counsel people regarding the Human Immunodeficiency Virus care pathway within the Unified Health System, from 52.6% to 94.7%.

**Conclusion:**

The participatory and dialogic health education program developed with nursing students proved effective in expanding knowledge about HIV and its care pathway, as well as in strengthening participants’ confidence in approaching people living with HIV.

## INTRODUCTION

The Human Immunodeficiency Virus (HIV) represents a major public health problem, as it affects a significant portion of the population and entails significant clinical, social, and economic consequences, exacerbating other health conditions and increasing challenges for care and prevention systems^(1)^. In Brazil, from 2007 to June 2023, 489, 594 new cases of HIV infection were reported, and when comparing the years 2020 and 2022, the number of HIV infection cases increased by 17.2% nationwide^(2)^.

Nurses play a central role in comprehensive HIV/AIDS care, working across the spectrum from prevention to the long-term follow-up of patients.

It is the responsibility of these professionals to facilitate access to and adherence to pre- and post-exposure prophylaxis for HIV (PrEP and PEP), offering these interventions in accordance with established criteria, as well as to conduct health education initiatives, perform rapid diagnostic tests, and monitor clinical and psychosocial aspects related to HIV and to individuals already receiving antiretroviral therapy (ART) ^(3)^.

Despite the importance of nurses’ role in this line of care, studies point to gaps in these professionals’ knowledge about HIV ^(4,5,6,7)^. This situation may be linked to the still-insufficient coverage of the topic during health education, which directly impacts the quality of care and can lead to individual and collective consequences, such as missing the window of opportunity for PEP and an increased risk of new infections^(8,9)^.

Thus, the quality of health education—understood as an educational, political, and social process that prepares professionals through various teaching and learning strategies^(10)^—constitutes, especially in nursing, a key element for strengthening public policies aimed at people living with HIV. By expanding technical knowledge and fostering a more sensitive understanding of the social, ethical, and subjective dimensions of care, this training contributes to more equitable professional practice, which brings users closer to the healthcare system and improves the quality of care provided.

Considering the importance of nurses’ role in the prevention and treatment of HIV/AIDS and the gap in the nursing education process, this study aimed to develop and evaluate a participatory and dialogic health education program with nursing students at a community college in the city of Criciúma, Santa Catarina, regarding the Human Immunodeficiency Virus and its care pathway.

## METHOD

### Type of Study

Action research was used as the methodology for this study, which focuses on proposing and implementing an intervention that modifies the reality presented by the researcher and participants in the observed context^(11)^. According to Thiollent, action-research generally involves identifying a collective problem, planning an action, involving the participants, implementing the intervention, evaluating the results, and jointly producing knowledge^(12)^.

In addition, the theoretical framework was based on the principles of Critical Pedagogy described by Paulo Freire, whose core focus lies in the manifestations of power relations and inequality, problematizing them within educational settings^(13)^. Knowledge is constructed collaboratively, with both parties assuming the roles of educator and learner through dialogue and the exchange of knowledge^(13)^.

The reporting of the study’s qualitative aspects was guided by the recommendations of the Consolidated Criteria for Reporting Qualitative Research (COREQ), translated and validated into Portuguese^(14)^. The study design and data collection were conducted by the first author, a nurse specializing in public health and a master’s student in Nursing, with prior experience in health education and HIV and practice in primary health care through a multiprofessional residency in public health. The researcher had no hierarchical or evaluative relationship with the participants, although she shared the university institutional setting with them. During the study, efforts were made to minimize potential biases by using standardized data collection instruments, systematically documenting the research stages, and conducting an integrated analysis of quantitative and qualitative data, in accordance with the principles of action research and critical pedagogy.

### Location, Population, Selection Criteria, and Sample Definition

The study took place at a community college in the city of Criciúma, Santa Catarina, Brazil. Inclusion criteria were students enrolled in the undergraduate nursing program between the 5th and 10th phases of the program at this institution, aged 18 or older; exclusion criteria were students who stated they did not have the time to participate in the training.

The eligible population consisted of 250 students, and all were invited to participate. Of these, 40 expressed interest in participating in the study. However, the final sample consisted of 19 participants, as the others were unable to participate in the planned stages of the research, constituting a convenience sample. This limitation was related to the organization of classes into day and evening sessions, as well as to the nature of the program, which is characterized by a significant number of students who balance their academic studies with work activities.

### Study Protocol

The study was organized into four stages: exploratory, planning, action, and evaluation^(11)^, which are summarized in Chart 1.

**Chart 1:** Stages of the design and development of HIV health education for nursing students – Criciúma, SC, Brazil, 2025.

Stage	Objective	Student participation	Strategies used	Freire’s rationale	Output
*Exploratory*	Identify training needs related to HIV	Students completed a self-administered diagnostic questionnaire	Google Forms Questionnaire	Thematic inquiry, seeking to understand the participants’ reality and needs	Gap Analysis on PrEP, PEP, ART, and Welcoming Services
*Planning*	Structure the training	Student feedback informed the planning process	Definition of competencies, objectives, and clinical cases	Questioning reality, transforming identified needs into themes that guide training	Training Plan
*Action*	Develop knowledge about the HIV care pathway	Active participation in groups	Interactive presentation, discussion of clinical cases, and creation of mind maps	Dialogue, shared construction of knowledge, and praxis	Collective Knowledge Building
*Evaluation*	Assess learning and the effectiveness of the training	Self-assessment and evaluation of the activity	Self-administered questionnaire	Critical reflection on practice and the reconstruction of knowledge	Identification of Strengths and Areas for Improvement

Source: Prepared by the authors, 2026.

The organization of health education was guided by the principles of Freirean critical pedagogy^(13)^, particularly thematic inquiry, problematization of reality, dialogicity, and praxis. These principles were incorporated into all stages of the action-research, from identifying the students’ needs to the critical evaluation of the intervention carried out.

During the exploratory phase, the first author personally extended the invitation in the classrooms of the selected classes, presenting the research proposal and inviting the students to participate. Next, a self-administered questionnaire created on the *Google Forms* platform was made available; it was accessed via a *QR code* and included questions focused on the participants’ self-perception regarding their understanding of the HIV care continuum—including PrEP, PEP, and ART—as well as the main difficulties they experienced in relation to the topic.

The responses obtained through the form constituted the initial assessment for the educational intervention and made it possible to identify the main training needs highlighted by the students. Among these, the following stood out: questions regarding the role of nursing in the HIV care continuum; the management of and guidance on PrEP, PEP, and ART; the provision of comprehensive and patient-centered care; and uncertainties regarding the clinical and educational approach to practical situations involving people living with HIV or at risk of infection.

Thus, the problem statement was developed jointly with the participants, seeking to ensure that the topic addressed their needs and perceptions, while also fostering reflections capable of driving the desired changes. Based on this initial assessment, the planning phase began, during which the competency to be achieved following the training was defined: to understand the role of nursing in the HIV care continuum in a comprehensive and holistic manner. Based on the responses to the questionnaire, the priority training content was defined, and it was decided to address four clinical cases covering situations related to prevention, follow-up, treatment, and a comprehensive approach to HIV care^(15)^.

Case studies were chosen because they serve as a pedagogical strategy that strengthens the health education process, since case analysis is a dynamic and interactive teaching strategy that has been shown to improve critical thinking and problem-solving skills^(16)^. In this format, students experience Freirean praxis, understood as the continuous movement between action, reflection, and new action, in which critical thinking about reality serves as the foundation for transforming it^(11)^.

The combination of dialogic presentations, discussion of clinical cases, mind-mapping, and small-group work was chosen for its alignment with the principles of action-research and Freirean critical pedagogy, promoting the examination of concrete situations, the exchange of experiences among participants, the collective construction of knowledge, and the development of critical reflection on nursing practice in HIV care.

During the action phase, the academic training session took place on a day previously agreed upon and scheduled between the researcher and the students, in a classroom provided by the university, lasting 4 hours. The session began with a brief presentation by the researcher on the distinction between HIV and AIDS, risk behaviors and key populations, the epidemiology of the infection, and the combined prevention mandala.

Afterward, the participants were divided into four groups. Each group was provided with a clinical case study accompanied by guiding questions, the combined prevention mandala, the HIV/AIDS notification form, and the prescription forms for PEP, PrEP, and ART, as well as poster board and pens to create a mind map.

The clinical cases revealed distinct realities that require nurses to demonstrate sensitivity and an ethical approach. In the first case, a young man sought help after unprotected sex, providing an opportunity to discuss PEP criteria and the importance of Welcoming without judgment. In the second case, a sex worker sought out the Basic Health Unit for testing, highlighting the need to ensure confidentiality, respect, and clear explanations about PEP and PrEP. The third case presented the story of a homeless man who injects drugs, living with HIV without treatment and exhibiting symptoms indicative of an opportunistic infection, which led to reflection on diagnosis and how to support his adherence to care. Finally, the fourth case presented a pregnant woman who discovered her HIV diagnosis during prenatal care, a situation that required guidance, emotional support, appropriate referrals, and a careful approach to involving her partner.

The clinical cases were authored by the researchers and developed based on epidemiological data on HIV in Brazil, current protocols from the Ministry of Health, and nursing practice in the care of people living with HIV.

In this phase, knowledge was constructed collectively, drawing on the students’ experiences, perceptions, and prior knowledge in resolving the clinical cases. The researcher acted as a mediator of these exchanges, guiding and deepening the discussions to foster critical reflection on the topic. Thus, the principles of action-research and critical pedagogy were incorporated, particularly with regard to the students’ active participation in the learning process.

On that same day, the final stage of the research was conducted, aimed not only at assessing the knowledge developed by the students but also at analyzing the academic training program itself and its effectiveness. To this end, a self-administered questionnaire was administered, designed to identify the effects of the educational intervention and evaluate the implemented activity.

The instrument consisted of three dimensions: organization and didactics of the training; learning and development; and applicability and impact. This stage made it possible to identify strengths and areas for improvement, allowing for adjustments to the training program before its implementation in other contexts or institutions.

The “organization and didactic methods of training” dimension of the questionnaire consisted of three statements: *the training content was presented clearly and comprehensibly; the methodology used was appropriate for the training objectives; and the teaching materials (presentations, texts, videos, etc.) were of good quality*.

The “learning and development” dimension, on the other hand, consisted of four questions*: How would you rate your knowledge of the indications, risk behaviors, duration of therapy, and side effects of Pre-Exposure Prophylaxis (PrEP)?; how would you rate your knowledge of the indications, risk behaviors, duration of treatment, and side effects of Post-Exposure Prophylaxis (PEP)?; how would you rate your knowledge of the indications, risk behaviors, duration of treatment, and side effects of Antiretroviral Therapy (ART)?;* and *do you feel prepared to receive and counsel individuals within the Unified Health System regarding the HIV care pathway?*.

Questions regarding self-perceived knowledge and preparedness to work in the HIV care pathway were assessed using a five-point Likert scale, ranging from 1 (very poor or completely unprepared) to 5 (very good or completely prepared). For the purpose of presenting the results, responses were grouped into three categories: very good/good, fair, and poor/very poor for questions related to knowledge; and fully prepared/prepared, neutral, and unprepared/completely unprepared for the question regarding readiness to receive and counsel people in the HIV care pathway.

Finally, the “applicability and impact” dimension consisted of two statements: “The training content is relevant to my academic practice and future professional work”; and “The topic addressed should be given greater emphasis in the nursing program’s curriculum.”

After completing the academic training, students were provided with instructional material containing a brief summary of the main points covered. This material was intended to serve as a reference during internships and clinical practice, facilitating the review of the content covered in the training.

Furthermore, the process was systematized and resulted in the development of an educational resource titled “Training Guide on the Role of Nurses in HIV/AIDS Care,” prepared by the authors. The resource provides a step-by-step overview of the health training program developed in this study, including its structure, content, and strategies used, to facilitate its replication at other universities and healthcare institutions. Thus, it aims to serve as a theoretical and practical resource for the training and qualification of nursing students and professionals in addressing the topic of HIV/AIDS.

### Data Analysis and Processing

The quantitative data collected from the questionnaires administered before and after the training were primarily organized in an Excel spreadsheet.

Statistical analyses were performed using *IBM SPSS Statistics* software. The variables were presented as absolute and relative frequencies. To compare the pre- and post-training assessments, the Wilcoxon test^(17)^ for paired samples was used, given the nonparametric distribution of the data. The significance level adopted was 5% (p < 0.05).

In addition, the qualitative data, derived from the open-ended questions, were subjected to the content analysis proposed by Bardin^(18)^, following the stages of pre-analysis, exploration of the material, and interpretation of the results. Initially, a thorough reading of the responses was conducted, followed by the identification of units of meaning related to the students’ perceptions of the training and of HIV education in undergraduate programs. Subsequently, the content was grouped by thematic similarity, resulting in the analytical categories presented in the results. To ensure the anonymity of the participants, excerpts from their statements were identified by the letter “e” followed by sequential numbering (e1, e2, e3…), corresponding to the order of participation in the study.

### Ethical Considerations

The study protocol was submitted to and cleared by the institution’s Research Ethics Committee, under Opinion No. 7.606.538 and CAAE No. 88937925.6.0000.0119. Students who agreed to participate in the study completed and signed the Informed Consent Form prior to the start of the research procedures. To ensure anonymity, each participant was identified by an alphanumeric code, designated as e1, e2, e3, and so on.

## RESULTS

The results are presented in two stages: the development of health education and the evaluation of health education.

The health education development phase was designed based on the needs identified by the students during the exploratory stage of the action-research. The data collected revealed gaps in knowledge regarding PrEP, PEP, ART, and the role of nurses in the HIV care continuum, as well as the perception that the topic was addressed only superficially during their undergraduate studies.

Based on this assessment, the core competency of the training program was defined as understanding the role of nursing in the HIV care continuum in a comprehensive and holistic manner. To achieve this competency, learning objectives were established focused on combined prevention, diagnosis, treatment, and welcoming people living with HIV.

The training was organized as a four-hour in-person session, structured around interactive presentations, discussion of clinical cases, and the collective creation of mind maps. Four clinical cases were developed, representing different contexts of HIV-related care, including situations involving the indication for PEP, the use of PrEP, adherence to antiretroviral treatment, and the care of pregnant women living with HIV.

As a result of the research, educational material titled “*Training Guide on the Role of Nurses in HIV/AIDS Care*” was developed, containing a systematic overview of the educational proposal, the content covered, and the pedagogical strategies used, with the potential for replication in educational institutions and health services^(19)^.

The sample consisted of 19 students, all of whom were in their 8th through 10th semesters of the nursing undergraduate program. The sample included 17 cisgender women and 2 cisgender men; 12 were between 20 and 30 years old, 5 between 31 and 40, and 2 between 41 and 50; 14 identified as single, 4 as married, and 1 as in a stable relationship.

Table 1 presents the results for the “learning and development” dimension, both before and after the training. Also within this dimension, the students’ statements revealed that, although the topic had already been addressed throughout the curriculum, this coverage was limited and lacked sufficient depth. These accounts were organized into the category “Superficial curricular treatment of the topic,” represented by the following statements: “Yes, superficially” (e4); “We didn’t go into depth” (e12); and “Yes, but just basic content” (e14).

**Table 1:** Students’ self-assessment of their knowledge regarding the HIV care pathway, comparing pre- and post-academic training, with absolute and relative frequencies – Criciúma, SC, Brazil, 2025. (n = 19).

Assessment question	Pre-training questionnaire n (%)	Post-training questionnaire n (%)	p-value (Wilcoxon test)
Assessment of Knowledge About PrEP			
Very good/Good	4 (21.1%)	19 (100%)	p < 0.001
Fair	13 (68.4%)	0 (0%)	
Poor/Very poor	2 (10.5%)	0 (0%)	
Assessment of Knowledge About PEP			
Very good/Good	3 (15.8%)	19 (100%)	p < 0.001
Fair	13 (68.4%)	0 (0%)	
Poor/Very poor	3 (15.8%)	0 (0%)	
Assessment of Knowledge About ART			
Very good/Good	2 (10.5%)	19 (100%)	p < 0.001
Fair	12 (63.2%)	0 (0%)	
Poor/Very poor	5 (26.3%)	0 (0%)	
Assessment of Readiness to Receive and Counsel Individuals on the HIV Care Pathway in the SUS			
Fully prepared/Prepared	10 (52.6%)	18 (94.7%)	p = 0.020
Neutral	8 (42.1%)	1 (5.3%)	
Unprepared/Totally unprepared	1 (5.3%)	0 (0%)	

Source: Prepared by the authors, 2026.

**Figure 1:**
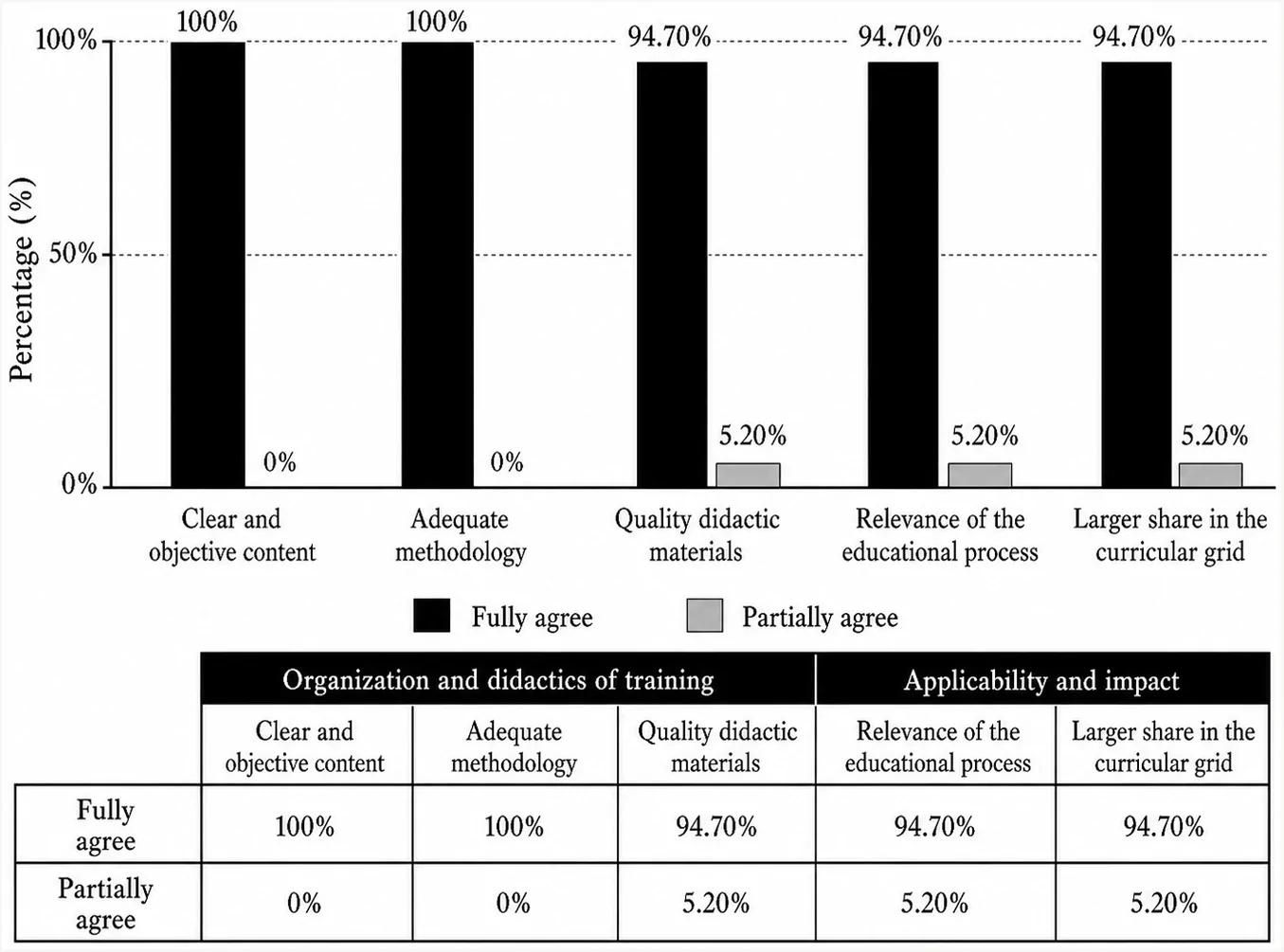
Evaluation of the training regarding organization, didactic methods, applicability, and impact – Criciúma, SC, Brazil, 2025. (n = 19).

Source: Prepared by the authors, 2026.

Figure 1 graphically presents the results for the dimensions “organization and didactics of training” and “applicability and impact,” collected after the training.

Finally, the category “Potential of Health Education” encompass students’ comments on the positive aspects of the activity. The reports indicate that the training stood out not only for the content covered but also for the way it was conducted, with an emphasis on the clarity of the explanations, the quality of the teaching methods, the proposed dynamics, and the active participation of the students. This perception is demonstrated in the following statements: “The teaching methods and approaches used” (e3); “I really liked the dynamics and the way the topics were addressed; it really held my attention” (e4); “The teaching methods presented, including discussion and case studies” (e11); “What I liked most about the training was the clear and up-to-date way of conveying knowledge that will be useful in professional practice” (e14); and “Above all, the participation of each student” (e15).

## DISCUSSION

Before the training began, the participants in this study rated their knowledge of PEP, PrEP, and ART, for the most part, as average. A similar result was observed in a study conducted with nursing students at a college in India, which assessed knowledge about HIV/AIDS, treatment, and prophylaxis, identifying moderate or low levels of understanding regarding the criteria for PEP prescription and the duration of its use, as well as the modes of transmission of the infection^(8)^. In the national context, studies also highlight gaps in knowledge regarding prophylaxis and care tailored to this specific population^(6,20,21)^.

In this regard, a study conducted with nurses in the capital of Santa Catarina state points to the limited coverage of the topic during undergraduate studies as one of the main factors contributing to insufficient knowledge^(22)^. While professionals already working in the field report not having received adequate training during their initial education, the student participants in this study confirm that, although the topic is covered in the classroom, it is addressed only superficially and briefly, with a greater focus on the biological aspects of the infection, without delving into the treatment and comprehensive care that should be provided to people living with HIV.

This scenario highlights an educational gap that can directly impact the clinical practice of professionals and students, as participants reported feelings of unpreparedness and insecurity when caring for people in the HIV care continuum. Another study also highlights this reality and reinforces that, for future professionals to provide guidance with confidence, welcoming attitude and responsibility, training must offer concrete learning opportunities on the subject^(23)^.

Both action-research and the critical and dialogic pedagogy proposed by Paulo Freire present themselves as powerful alternatives for working within the HIV care continuum, as they promote the construction of knowledge in a collaborative, horizontal, and contextualized manner, based on the analysis of real-life situations and dialogue.

In this sense, both align with the concept of liberating education by breaking with the “banking” model of teaching and fostering the development of critical, autonomous individuals capable of intervening in reality^(11)^.

The application of the Freirean method in this training enabled students to take on an active role in the learning process, moving beyond the passive position of content recipients to recognize themselves as critical, reflective individuals who share responsibility for the construction of knowledge. The dialogue established between the researcher and the students fostered the exchange of experiences and knowledge, valuing the participants’ prior knowledge and promoting critical reflection on the role of nurses in the face of social inequalities and the stigma associated with HIV.

In this context, the discussion expanded to include the ethical and political education of nurses, shifting the focus from the disease alone to the individual living with HIV, taking into account their history, context, and rights. Thus, the training also served as a space for raising awareness and empowerment, contributing to the repoliticization of health education—an essential aspect for advocating for comprehensive care and combating HIV-related stigma. By strengthening these future professionals’ ethical and political commitment to life and human rights, the experience reaffirms the role of liberating education in building more humane, critical, and socially committed care practices^(24)^.

The training proved to be effective and well-received by the students, as evidenced by the evaluation results, in which 100% of the participants rated the methodology used as appropriate for the proposed objectives. In addition, some students highlighted the didactic approach as one of the training’s strengths, as it fostered group participation and the exchange of knowledge. From the researcher’s perspective, the participants’ engagement was also fundamental to the successful conduct of the activity.

In addition to the students’ engagement, the training yielded important results regarding the participants’ self-perception of their knowledge related to HIV care and their professional preparedness to provide care to people in this context. After the training, all participants began to rate their knowledge of PrEP, PEP, and ART as “very good/good,” corresponding to 19 students (100%) in each of these areas, with a statistically significant difference compared to the pre-training period (p < 0.001). In addition, a significant improvement was observed in the perception of preparedness to welcome and counsel people regarding the HIV care pathway within the SUS, since 18 participants (94.7%) reported feeling “fully prepared/prepared” after the training, compared to 10 (52.6%) before the intervention, with statistical significance according to the Wilcoxon test (p = 0.020).

Thus, these findings may be related to the critical reflection fostered by the discussions of clinical cases, which enabled the integration of action and reflection, promoting a critical internalization of the content rather than mere memorization^(11,13)^. By sharing questions, experiences, and interpretations during the collective analysis of clinical cases, the students not only understood the content covered but also began to recognize their own potential for intervention in patient care, strengthening their ethical and political commitment to a more humane, holistic, and socially engaged practice.

A study conducted with 63 nurses also used a similar methodology for training, through group discussions and the collective construction of knowledge. The findings concur with the results presented here, as participants reported that the training was not limited to technical concepts but also sought to foster critical awareness of the social and cultural determinants of health^(25)^.

Therefore, the results may act as catalysts for improving curricular strategies in nursing programs. By demonstrating that the dialogic analysis of clinical cases strengthens students’ autonomy, critical thinking, and confidence, the findings point to the need to expand pedagogical practices that bridge the gap between theory and the reality of care. The ethical focus in clinical simulations and case studies promotes compassionate and critical decision-making, especially in contexts marked by stigma, such as care for people living with HIV^(24)^.

Finally, it is worth noting that training critical professionals committed to strengthening the SUS (Brazilian Unified Health System) constitutes an essential part of the social role of community colleges. In Santa Catarina, these institutions represent a significant and historically relevant reality in the training of health workers. By assuming this commitment, the community colleges strengthen not only the quality of education but also the development of more humane, comprehensive, and socially responsible care practices.

This study has some limitations that should be considered when interpreting the results. Although the training was aimed at students in phases 5 through 10, only students in phases 8 through 10 participated. This was due to the curriculum organization of the other phases, which had classes scheduled at the same time as the training, making their participation impossible.

The study was conducted with three classes at a single university, which may limit the generalizability of the findings to other institutions, regions, or contexts. Although the sample was adequate for the proposed objectives, its size was relatively small. Furthermore, it should be noted that the instruments used were based on self-assessment, reflecting the participants’ perceptions of their knowledge and preparedness to work in HIV care, and did not constitute an objective measure of performance or knowledge acquisition.

Similarly, the qualitative results are derived from the experiences reported by the participants and the interpretation carried out by the researchers, and are subject to the particularities of the context under investigation and the characteristics inherent to qualitative research.

Despite these limitations, this study highlights the potential of training designed in a participatory and dialogic manner. The experience demonstrated that, even with a small group, it was possible to expand knowledge, strengthen students’ confidence, and bring them closer to the reality of caring for people living with HIV. The integration of theory and practice, through contextualized clinical cases, fostered meaningful learning aligned with the principles of the SUS, indicating that active methodologies can have a significant impact on the educational process and be adapted to different health education contexts.

## CONCLUSION

The participatory and dialogic health education program developed with nursing students at a community college in Criciúma, Santa Catarina, proved effective in expanding knowledge about HIV and its care pathway, as well as in strengthening participants’ confidence when interacting with people living with HIV.

The findings show that the identified gaps are not limited to technical knowledge regarding PrEP, PEP, and ART, but also involve ethical, social, and subjective dimensions of care. In this regard, the educational experience reinforces the importance of more intentionally incorporating active methodologies and spaces for dialogue into nursing education, thereby fostering the development of professionals who are more critical, sensitive to stigma, and committed to comprehensive care within the Brazilian Unified Health System (SUS).

Thus, this study contributes by proposing an educational experience that demystifies not only HIV but also the way this topic is taught, promoting more humane pedagogical practices aligned with comprehensive health care.

## DATA AVAILABILITY

The entire dataset supporting the results of this study was published in the article itself.
