# Supplementary material for: Demystifying HIV: academic training for undergraduate nursing students
Source: Rev Esc Enferm USP. 2026 Sep 7;60:e20260023. doi: 10.1590/1980-220X-REEUSP-2026-0023en (PMC13561535; doi:10.1590/1980-220X-REEUSP-2026-0023en)
Supplement: Translation

# Translation: Desmistificando o HIV: formação acadêmica para estudantes da graduação em enfermagem

**DOI:** 10.1590/1980-220X-REEUSP-2026-0023pt

**Authors:** Naomi Tamima Estevam Cipriano, Jacks Soratto, Cristiane Damiani Tomasi

**Affiliations:** 1 Universidade Federal de Santa Catarina, Florianópolis, SC, Brasil.; 2 Universidade do Extremo Sul Catarinense, Criciúma, SC, Brasil.

**Keywords:** HIV, Cuidados de Enfermagem, Educação em Enfermagem, Aprendizagem Baseada em Problemas

## Abstract

**Objetivo:**

Desenvolver e avaliar uma formação em saúde, de caráter participativo e dialógico, com estudantes do curso de enfermagem de uma universidade comunitária de Criciúma/SC, sobre o Vírus da Imunodeficiência Humana e sua linha de cuidado.

**Método:**

Pesquisa-ação, fundamentada na Pedagogia Crítica, realizada com 19 estudantes de enfermagem entre a 8ª e a 10ª fase do curso. A formação ocorreu em quatro etapas: exploratória, planejamento, ação e avaliação, com questionários antes e após a intervenção.

**Resultados:**

Antes da formação, predominavam autoavaliações regulares ou ruins/muito ruins sobre Profilaxia Pré-Exposição, Profilaxia Pós-Exposição e Terapia Antirretroviral. Após a intervenção, todos classificaram seus conhecimentos como muito bom/bom. Também houve aumento da percepção de preparo para acolher e orientar pessoas sobre a linha de cuidado do Vírus da Imunodeficiência Humana no Sistema Único de Saúde, de 52,6% para 94,7%.

**Conclusão:**

A formação em saúde participativa e dialógica desenvolvida com estudantes de enfermagem mostrou-se efetiva para ampliar conhecimentos sobre o HIV e sua linha de cuidado, bem como para fortalecer a segurança dos participantes na abordagem às pessoas vivendo com HIV.

## INTRODUÇÃO

O Vírus da Imunodeficiência Humana (HIV) representa um importante problema de saúde pública, pois atinge parcela significativa da população e acarreta consequências clínicas, sociais e econômicas relevantes, potencializando outros agravos à saúde e ampliando desafios para os sistemas de cuidado e prevenção^(1)^. No Brasil, no período de 2007 a junho de 2023, 489.594 novos casos de infecção por HIV foram notificados, e comparando os anos de 2020 e 2022, o número de casos de infecção pelo vírus aumentou 17,2% no país^(2)^.

O enfermeiro desempenha papel central no cuidado integral em HIV/AIDS, atuando desde a prevenção até o acompanhamento longitudinal das pessoas atendidas. Cabe a esse profissional facilitar o acesso e a adesão às profilaxias pré e pós-exposição ao HIV (PrEP e PEP), ofertando essas tecnologias conforme os critérios estabelecidos, além de desenvolver ações de educação em saúde, realizar testes rápidos para diagnóstico e acompanhar aspectos clínicos e psicossociais relacionados ao HIV e às pessoas que já fazem uso da Terapia antirretroviral (TARV)^(3)^.

Apesar da importância da atuação do enfermeiro nesta linha de cuidado, estudos apontam fragilidades no conhecimento desses profissionais sobre o HIV^(4,5,6,7)^. Essa realidade pode estar associada à abordagem ainda insuficiente da temática durante a formação em saúde, o que repercute diretamente na qualidade da assistência e pode gerar consequências individuais e coletivas, como a perda da janela de tempo para o uso da PEP e aumento do risco de novas infecções^(8,9)^.

Dessa forma, a qualificação da formação em saúde, compreendida como um processo educativo, político e social que prepara profissionais por meio de diferentes estratégias de ensino e aprendizagem^(10)^ constitui, especialmente na enfermagem, um elemento importante para o fortalecimento das políticas públicas voltadas às pessoas vivendo com HIV. Ao ampliar o conhecimento técnico e favorecer uma compreensão mais sensível das dimensões sociais, éticas e subjetivas do cuidado, a formação contribui para uma atuação profissional mais equitativa, o que aproxima os usuários da rede de atenção e qualifica o acompanhamento ofertado.

Considerando a importância da atuação do enfermeiro na prevenção e tratamento do HIV/AIDS e a lacuna no processo formativo de enfermeiros, o presente estudo objetivou desenvolver e avaliar uma formação em saúde, de caráter participativo e dialógico, com alunos do curso de enfermagem de uma universidade comunitária da cidade de Criciúma/SC, a respeito do Vírus da Imunodeficiência Humana e sua linha de cuidado.

## MÉTODO

### Tipo do Estudo

Utilizou-se da pesquisa-ação como metodologia deste estudo, cuja direciona seus esforços para a proposição e aplicação de uma intervenção que modifique a realidade apresentada pelo pesquisador e participantes do contexto observado^(11)^. Para Thiollent, a pesquisa-ação envolve, de modo geral, a identificação de um problema coletivo, o planejamento de uma ação, a participação dos sujeitos envolvidos, a implementação da intervenção, a avaliação dos resultados e a produção conjunta de conhecimento^(12)^.

Conjuntamente, foi utilizado embasamento teórico a partir dos princípios da Pedagogia Crítica descritos por Paulo Freire, cujo cerne apresenta-se como as manifestações das relações de poder e desigualdade, problematizando-as nos espaços de educação^(13)^. O saber é construído de forma conjunta, no qual ambas as partes ocupam posições de educador e educando, através do diálogo e trocas de conhecimentos^(13)^.

O relato dos aspectos qualitativos do estudo foi orientado pelas recomendações do Consolidated Criteria for Reporting Qualitative Research (COREQ), traduzido e validado para a língua portuguesa^(14)^. A formação e a coleta de dados foram conduzidas pela primeira autora, enfermeira especialista em saúde coletiva e mestranda em Enfermagem, com experiência prévia em educação em saúde e HIV e atuação na atenção primária em saúde por meio da residência multiprofissional em saúde coletiva. A pesquisadora não possuía relação hierárquica ou avaliativa com os participantes, embora compartilhasse com eles o ambiente institucional universitário. Durante o desenvolvimento do estudo, buscou-se minimizar possíveis vieses por meio da utilização de instrumentos padronizados para coleta de dados, registro sistemático das etapas da pesquisa e análise articulada entre os dados quantitativos e qualitativos, respeitando os pressupostos da pesquisa-ação e da pedagogia crítica.

### Local, População, Critérios de Seleção e Definição da Amostra

O estudo aconteceu em uma Universidade Comunitária da cidade de Criciúma, Santa Catarina, Brasil, tendo como critérios de inclusão alunos matriculados na graduação em enfermagem entre a 5ª e a 10ª fase do curso desta universidade, com idade acima de 18 anos; e como critérios de exclusão os alunos que declararam não apresentar disponibilidade de tempo para participar da formação.

A população elegível era composta por 250 estudantes, e o convite para participação foi realizado a todos. Desses, 40 manifestaram interesse em participar do estudo. No entanto, a amostra final foi constituída por 19 participantes, uma vez que os demais não apresentaram disponibilidade para participar das etapas previstas na pesquisa, caracterizando uma amostragem por conveniência. Essa limitação esteve relacionada à organização das turmas em períodos diurno e noturno, bem como ao perfil do curso, caracterizado por uma quantidade expressiva de estudantes que conciliam a formação acadêmica com atividades laborais.

### Protocolo do Estudo

O estudo foi organizado em 4 etapas: exploratória, planejamento, ação e avaliação^(11)^, que estão sintetizadas no Quadro 1.

**Quadro 1:** Etapas da construção e desenvolvimento da formação em saúde sobre HIV para estudantes de enfermagem – Criciúma, SC, Brasil, 2025.

Etapa	Objetivo	Participação dos estudantes	Estratégias utilizadas	Fundamentação freiriana	Produto gerado
*Exploratória*	Identificar necessidades formativas sobre HIV	Responderam questionário diagnóstico autoadministrado	Questionário Google Forms	Investigação temática, buscando compreender a realidade e as necessidades dos participantes	Levantamento de lacunas sobre PrEP, PEP, TARV e acolhimento
*Planejamento*	Estruturar a formação	Demandas dos estudantes subsidiaram o planejamento	Definição de competência, objetivos e casos clínicos	Problematização da realidade, transformando necessidades identificadas em temas geradores para a formação	Plano da formação
*Ação*	Desenvolver conhecimentos sobre a linha de cuidado do HIV	Participação ativa em grupos	Exposição dialogada, discussão de casos clínicos, construção de mapas mentais	Diálogo, construção compartilhada do conhecimento e práxis	Construção coletiva do conhecimento
*Avaliação*	Avaliar aprendizagem e efetividade da formação	Autoavaliação e avaliação da atividade	Questionário autoadministrado	Reflexão crítica sobre a prática e reconstrução do conhecimento	Identificação de potencialidades e aspectos a aprimorar

Fonte: Elaborado pelos autores, 2026.

A organização da formação em saúde foi orientada pelos pressupostos da pedagogia crítica de freiriana^(13)^, especialmente pela investigação temática, problematização da realidade, dialogicidade e práxis. Esses pressupostos foram incorporados em todas as etapas da pesquisa-ação, desde a identificação das necessidades dos estudantes até a avaliação crítica da intervenção realizada.

Na fase exploratória, a primeira autora realizou o convite presencialmente nas salas de aula das turmas selecionadas, apresentando a proposta da pesquisa e convidando os estudantes à participação. Em seguida, foi disponibilizado um questionário autoadministrado elaborado na plataforma *Google Forms*, acessado por meio de *QR Code*, com questões voltadas à autopercepção dos participantes sobre seu entendimento acerca da linha de cuidado do HIV, incluindo PrEP, PEP e TARV, bem como às principais dificuldades vivenciadas em relação à temática.

As respostas obtidas por meio do formulário constituíram o diagnóstico inicial da intervenção educativa e permitiram identificar as principais necessidades formativas apontadas pelos estudantes. Entre elas, destacaram-se dúvidas relacionadas à atuação da enfermagem na linha de cuidado do HIV, ao manejo e orientação sobre PrEP, PEP e TARV, à condução do cuidado de forma integral e humanizada, além de inseguranças quanto à abordagem clínica e educativa diante de situações práticas envolvendo pessoas vivendo com HIV ou em risco de infecção.

Dessa forma, o problema foi construído em conjunto com os participantes, buscando garantir que a temática dialogasse com suas necessidades e percepções, além de favorecer reflexões capazes de impulsionar as transformações desejadas. A partir desse diagnóstico inicial, iniciou-se a etapa de planejamento, na qual foi estipulada a competência a ser alcançada após a aplicação da formação: compreender a atuação da enfermagem na linha de cuidado do HIV de forma integral e holística. Com base nas respostas do formulário, foram definidos os conteúdos prioritários da formação e optou-se pela resolução de quatro casos clínicos, contemplando situações relacionadas à prevenção, ao acompanhamento, ao tratamento e à abordagem integral no cuidado em HIV^(15)^.

Optou-se pela utilização de estudos de caso uma vez que estes se apresentam como estratégia pedagógica que fortalecem o processo de formação em saúde, uma vez que a análise de casos é uma estratégia de ensino dinâmica e interativa que apresenta melhorias nas habilidades de pensamento crítico e resolução de problemas^(16)^. Nesse formato, os estudantes vivenciam a práxis freiriana, entendida como o movimento contínuo entre ação, reflexão e nova ação, no qual o pensar crítico sobre a realidade serve de fundamento para transformá-la^(11)^. A combinação entre exposição dialogada, discussão de casos clínicos, construção de mapas mentais e trabalho em pequenos grupos foi definida por sua consonância com os pressupostos da pesquisa-ação e da pedagogia crítica freiriana, favorecendo a problematização de situações concretas, a troca de experiências entre os participantes, a construção coletiva do conhecimento e o desenvolvimento da reflexão crítica acerca da atuação da enfermagem na linha de cuidado do HIV.

Na fase da ação, ocorreu a aplicação da formação acadêmica em um dia previamente acordado e agendado entre a pesquisadora e os acadêmicos, em uma sala disponibilizada pela universidade, com duração de 4 horas. O encontro iniciou-se com uma breve explanação da pesquisadora sobre a diferenciação do HIV e da AIDS, os comportamentos de risco e populações chave, a epidemiologia da infecção e a mandala de prevenção combinada.

Após, os participantes foram divididos em 4 grupos, sendo disponibilizado um caso clínico com questões norteadoras para cada grupo, a mandala da prevenção combinada, a ficha de notificação de HIV/AIDS e os formulários de prescrição da PEP, PrEP e TARV; bem como cartolina e canetas para confecção de um mapa mental.

Os casos clínicos revelaram realidades distintas que exigem do enfermeiro sensibilidade e postura ética. No primeiro, um jovem buscou ajuda após uma relação sexual desprotegida, abrindo espaço para conversar sobre os critérios da PEP e a importância de um acolhimento sem julgamentos. No segundo, uma trabalhadora do sexo procurou a Unida Básica de Saúde para testagem, mostrando a necessidade de garantir sigilo, respeito e esclarecimentos sobre PEP e PrEP. O terceiro caso trouxe a história de um homem em situação de rua e usuário de drogas injetáveis, vivendo com HIV sem tratamento e com sintomas indicativos de infecção oportunista, o que levou à reflexão sobre diagnóstico e sobre como apoiar sua adesão ao cuidado. Por fim, o quarto caso apresentou uma gestante que descobriu o diagnóstico de HIV durante o pré-natal, situação que demandou orientação, apoio emocional, encaminhamentos adequados e a abordagem cuidadosa do parceiro.

Os casos clínicos foram de autoria das pesquisadoras e elaborados com base nos dados epidemiológicos do HIV no Brasil, nos protocolos vigentes do Ministério da Saúde e na prática da enfermagem no cuidado às pessoas que vivem com HIV.

Nesta fase, o conhecimento foi construído de forma coletiva, a partir das vivências, percepções e conhecimentos prévios dos estudantes na resolução dos casos clínicos. A pesquisadora atuou como mediadora das trocas, conduzindo e aprofundando as discussões de modo a favorecer a reflexão crítica sobre a temática. Assim, foram incorporados os princípios da pesquisa-ação e da pedagogia crítica, especialmente no que se refere à participação ativa dos estudantes no processo de aprendizagem.

Neste mesmo dia, foi realizada a última etapa da pesquisa, destinada não apenas à avaliação dos conhecimentos desenvolvidos pelos estudantes, mas também à análise da própria formação acadêmica e de sua efetividade. Para isso, foi aplicado um questionário autoadministrado, elaborado para identificar os efeitos da ação educativa e avaliar a atividade implementada. O instrumento foi composto por três dimensões: organização e didática da formação; aprendizagem e desenvolvimento; e aplicabilidade e impacto. Essa etapa permitiu identificar potencialidades e aspectos a serem aprimorados, possibilitando ajustes na formação antes de sua aplicação em outros contextos ou instituições.

A dimensão “organização e didática da formação” do questionário era composta por 3 afirmações: *o conteúdo da formação foi apresentado de forma clara e compreensível; a metodologia utilizada foi adequada aos objetivos de formação; e os materiais didáticos (apresentações, textos, vídeos, etc.) foram de boa qualidade*.

Já a dimensão “aprendizagem e desenvolvimento” era composta por 4 questionamentos*: como você avaliaria seu conhecimento sobre as indicações, comportamentos de risco, tempo de duração da terapêutica e efeitos colaterais da Profilaxia Pré-exposição (PrEP)?; como você avaliaria seu conhecimento sobre as indicações, comportamentos de risco, tempo de duração da terapêutica e efeitos colaterais da Profilaxia Pós-exposição (PEP)?; como você avaliaria seu conhecimento sobre as indicações, comportamentos de risco, tempo de duração da terapêutica e efeitos colaterais da Terapia Antirretroviral (TARV)?;* e *você se sente preparado(a) para acolher e orientar pessoas no Sistema Único de Saúde acerca da linha de cuidado do HIV?*.

As questões referentes à autopercepção do conhecimento e ao preparo para atuação na linha de cuidado do HIV foram avaliadas por meio de escala Likert de cinco pontos, variando de 1 (muito ruim ou totalmente despreparado) a 5 (muito bom ou totalmente preparado). Para fins de apresentação dos resultados, as respostas foram agrupadas em três categorias: muito bom/bom, regular e ruim/muito ruim para as questões relacionadas ao conhecimento; e totalmente preparado/preparado, neutro e despreparado/totalmente despreparado para a questão referente ao preparo para acolher e orientar pessoas na linha de cuidado do HIV.

Por fim, a dimensão “aplicabilidade e impacto”, era composta por 2 afirmações: o conteúdo da formação é relevante para minha prática acadêmica e futura atuação profissional; e a temática abordada deveria estar mais presente na grade curricular do curso de enfermagem.

Após a realização da formação acadêmica, foi disponibilizado aos estudantes um material didático contendo um breve resumo dos principais pontos abordados. Esse material teve como objetivo servir como apoio para consulta durante os estágios e as práticas clínicas, favorecendo a retomada dos conteúdos trabalhados na formação.

Ainda, o processo foi sistematizado e resultou na construção de um material educativo intitulado “Guia de formação sobre a atuação do enfermeiro na linha de cuidado do HIV/AIDS”, elaborado pelos autores. O material apresenta o passo a passo da formação em saúde desenvolvida no estudo, incluindo sua organização, conteúdos abordados e estratégias utilizadas, de modo a possibilitar sua replicação em outras universidades e instituições de saúde. Dessa forma, busca servir como suporte teórico-prático para a formação e qualificação de estudantes e profissionais de enfermagem na abordagem da temática do HIV/AIDS.

### Análise e Tratamento dos Dados

Os dados quantitativos coletados nos questionários aplicados antes e após a formação foram organizados primariamente em uma tabela no Excel. As análises estatísticas foram realizadas utilizando o *software IBM SPSS Statistics*. As variáveis foram apresentadas em frequências absolutas e relativas. Para comparar as avaliações antes e após a formação, utilizou-se o teste de Wilcoxon^(17)^ para amostras pareadas, considerando a distribuição não paramétrica dos dados. O nível de significância adotado foi de 5% (p < 0,05).

Além disso, os dados qualitativos, oriundos das perguntas abertas, foram submetidos à análise de conteúdo proposta por Bardin^(18)^, seguindo as etapas de pré-análise, exploração do material e interpretação dos resultados. Inicialmente, realizou-se leitura exaustiva das respostas, seguida da identificação de unidades de sentido relacionadas às percepções dos estudantes sobre a formação e sobre o ensino do HIV na graduação. Posteriormente, os conteúdos foram agrupados por similaridade temática, originando as categorias analíticas apresentadas nos resultados. Para garantir o anonimato dos participantes, os excertos das falas foram identificados pela letra “e” seguida de numeração sequencial (e1, e2, e3…), correspondente à ordem de participação no estudo.

### Aspectos Éticos

O projeto deste estudo foi submetido e aprovado pelo Comitê de Ética em Pesquisa da instituição, sob parecer nº 7.606.538 e CAAE nº 88937925.6.0000.0119. Os alunos que aceitaram participar da pesquisa realizaram o preenchimento e a assinatura do Termo de Consentimento Livre e Esclarecido previamente ao início dos procedimentos de pesquisa. Para garantir o anonimato, cada participante foi identificado por meio de um código alfanumérico, sendo designados como e1, e2, e3, e assim sucessivamente.

## RESULTADOS

Os resultados são apresentados em duas etapas, a de desenvolvimento da formação em saúde e a de avaliação da formação em saúde.

A fase de desenvolvimento da formação em saúde foi construída a partir das necessidades identificadas pelos estudantes na etapa exploratória da pesquisa-ação. Os dados levantados evidenciaram lacunas relacionadas ao conhecimento sobre PrEP, PEP, TARV e à atuação do enfermeiro na linha de cuidado do HIV, além da percepção de abordagem superficial da temática durante a graduação.

Com base nesse diagnóstico, definiu-se como competência central da formação compreender a atuação da enfermagem na linha de cuidado do HIV de forma integral e holística. Para alcançar essa competência, foram estabelecidos objetivos de aprendizagem voltados à prevenção combinada, ao diagnóstico, ao tratamento e ao acolhimento das pessoas vivendo com HIV.

A formação foi organizada em uma sessão presencial com duração de quatro horas, estruturada a partir de exposição dialogada, discussão de casos clínicos e construção coletiva de mapas mentais. Foram elaborados quatro casos clínicos representando diferentes contextos de cuidado relacionados ao HIV, contemplando situações envolvendo indicação de PEP, utilização de PrEP, adesão ao tratamento antirretroviral e acompanhamento de gestantes vivendo com HIV.

Como produto da pesquisa, foi desenvolvido o material educativo intitulado “*Guia de formação sobre a atuação do enfermeiro na linha de cuidado do HIV/AIDS*”, contendo a sistematização da proposta educativa, os conteúdos trabalhados e as estratégias pedagógicas utilizadas, com potencial de replicação em instituições de ensino e serviços de saúde^(19)^.

A amostragem totalizou 19 alunos, estando todos entre a 8ª e a 10ª fase da graduação em enfermagem. Foram incluídas 17 mulheres cisgênero e 2 homens cisgênero; 12 com idade entre 20 e 30 anos, 5 entre 31 e 40 anos e 2 entre 41 e 50 anos; 14 declararam-se solteiros, 4 casados e 1 em união estável.

A Tabela 1 apresenta os resultados da dimensão “aprendizagem e desenvolvimento”, pré e pós-aplicação da formação. Ainda nessa dimensão, as falas dos acadêmicos evidenciaram que, embora a temática já tivesse sido abordada ao longo da grade curricular, essa abordagem ocorreu de maneira limitada, sem aprofundamento suficiente. Esses relatos foram organizados na categoria “Abordagem curricular superficial da temática”, representada pelas seguintes falas: “Sim, superficialmente” (e4); “Não nos aprofundamos” (e12); e “Sim, mas conteúdo básico” (e14).

**Tabela 1:** Autoavaliação dos alunos acerca de seus conhecimentos frente a linha de cuidado do HIV, comparativo entre pré e pós-aplicação da formação acadêmica, frequência absoluta e relativa – Criciúma, SC, Brasil, 2025. (n = 19).

Questão avaliativa	Questionário pré-formação n (%)	Questionário pós-formação n (%)	p-valor (teste de Wilcoxon)
Avaliação do conhecimento sobre PrEP			
Muito bom/Bom	4 (21,1%)	19 (100%)	p < 0,001
Regular	13 (68,4%)	0 (0%)	
Ruim/Muito ruim	2 (10,5%)	0 (0%)	
Avaliação do conhecimento sobre PEP			
Muito bom/Bom	3 (15,8%)	19 (100%)	p < 0,001
Regular	13 (68,4%)	0 (0%)	
Ruim/Muito ruim	3 (15,8%)	0 (0%)	
Avaliação do conhecimento sobre TARV			
Muito bom/Bom	2 (10,5%)	19 (100%)	p < 0,001
Regular	12 (63,2%)	0 (0%)	
Ruim/Muito ruim	5 (26,3%)	0 (0%)	
Avaliação do preparo para acolher e orientar pessoas sobre a linha de cuidado do HIV no SUS			
Totalmente preparado/Preparado	10 (52,6%)	18 (94,7%)	p = 0,020
Neutro	8 (42,1%)	1 (5,3%)	
Despreparado/Totalmente despreparado	1 (5,3%)	0 (0%)	

Fonte: Elaborado pelos autores, 2026.

A Figura 1 apresenta graficamente os resultados das dimensões “organização e didática da formação” e “aplicabilidade e impacto”, coletados pós-formação.

**Figura 1:** Avaliação da formação quanto à organização, didática, aplicabilidade e impacto – Criciúma, SC, Brasil, 2025. (n = 19).

Fonte: Elaborado pelos autores, 2026.

Por fim, a categoria “Potencialidades da formação em saúde” reúne as falas dos estudantes sobre os aspectos positivos da atividade. Os relatos indicam que a formação se destacou não apenas pelo conteúdo abordado, mas também pela forma como foi conduzida, com ênfase na clareza das explicações, na qualidade da didática, na dinâmica proposta e na participação ativa dos acadêmicos. Essa percepção é demonstrada nas falas: “A didática e os métodos utilizados” (e3); “Gostei muito da dinâmica e da forma que abordou os assuntos, me prendeu bastante” (e4); “Da didática apresentada, discussão e estudo de caso” (e11); “O que eu mais gostei na formação foi a forma clara e atualizada de transmitir conhecimentos que serão úteis na prática profissional” (e14); e “Além de tudo, a participação de cada estudante” (e15).

## DISCUSSÃO

Os participantes da presente pesquisa avaliaram, antes da aplicação da formação, seus conhecimentos sobre PEP, PrEP e TARV, em sua maioria, como regulares. Resultado semelhante foi observado em uma pesquisa realizada com estudantes de enfermagem de uma faculdade na Índia, que avaliou o conhecimento sobre HIV/AIDS, tratamento e profilaxias, identificando níveis regulares ou baixos de compreensão sobre os critérios de indicação e o tempo de uso da PEP, bem como sobre as formas de transmissão da infecção^(8)^. No contexto nacional, estudos também evidenciam lacunas no conhecimento sobre profilaxias e cuidados direcionados a essa população específica^(6,20,21)^.

Nesse sentido, uma pesquisa realizada com enfermeiros da capital de Santa Catarina aponta a pouca abordagem da temática durante a graduação como um dos principais fatores relacionados à insuficiência de conhecimento^(22)^. Enquanto profissionais já inseridos no mercado relatam não terem recebido subsídios adequados durante sua formação inicial, os estudantes participantes deste estudo confirmam que, embora o tema seja abordado em sala de aula, isso ocorre de maneira superficial e rápida, com maior enfoque nos aspectos biológicos da infecção, sem aprofundamento sobre o tratamento e o cuidado integral que deve ser oferecido às pessoas que vivem com HIV.

Esse cenário evidencia uma lacuna formativa que pode impactar diretamente a prática clínica de profissionais e acadêmicos, uma vez que os participantes relataram sentimentos de despreparo e insegurança para atender pessoas inseridas na linha de cuidado do HIV. Outro estudo também aponta essa realidade e reforça que, para que futuros profissionais possam orientar com segurança, acolhimento e responsabilidade, é necessário que a formação ofereça oportunidades concretas de aprendizagem sobre a temática^(23)^.

Tanto a pesquisa-ação quanto a pedagogia crítica e dialógica proposta por Paulo Freire apresentam-se como alternativas potentes para o trabalho com a linha de cuidado do HIV, por favorecerem a construção do conhecimento de forma conjunta, horizontal e contextualizada, a partir da problematização de situações reais e do diálogo. Nesse sentido, ambas se alinham ao conceito de educação libertadora, ao romperem com o modelo bancário de ensino e favorecerem a formação de sujeitos críticos, autônomos e capazes de intervir na realidade^(11)^.

A aplicação do método freiriano nesta formação possibilitou que os estudantes assumissem papel ativo no processo de aprendizagem, deixando a posição passiva de receptores de conteúdo para se reconhecerem como sujeitos críticos, reflexivos e corresponsáveis pela construção do conhecimento. O diálogo estabelecido entre pesquisadora e estudantes favoreceu a troca de experiências e saberes, valorizando os conhecimentos prévios dos participantes e promovendo uma reflexão crítica sobre o papel do enfermeiro diante das desigualdades sociais e da estigmatização associada ao HIV.

Nesse movimento, a discussão ampliou-se para a formação ética e política do enfermeiro, deslocando o foco exclusivamente da doença para o sujeito que vive com HIV, considerando sua história, seu contexto e seus direitos. Assim, a formação configurou-se também como um espaço de conscientização e empoderamento, contribuindo para a repolitização da formação em saúde, aspecto essencial para a defesa da integralidade do cuidado e para o enfrentamento do estigma relacionado ao HIV. Ao fortalecer o compromisso ético-político dessesfuturos profissionais com a vida e os direitos humanos, a experiência reafirma o papel da educação libertadora na construção de práticas de cuidado mais humanas, críticas e socialmente comprometidas^(24)^.

A formação demonstrou-se efetiva e bem aceita pelos estudantes, conforme evidenciado pelos resultados da avaliação, em que 100% dos participantes classificaram a metodologia utilizada como adequada aos objetivos propostos. Além disso, alguns estudantes destacaram a didática como um dos pontos fortes da capacitação, por favorecer a participação do grupo e a troca de saberes. Do ponto de vista da pesquisadora, o engajamento dos participantes também foi fundamental para o bom desenvolvimento da atividade.

Além do engajamento dos estudantes, a formação apresentou resultados importantes quanto à autopercepção dos participantes sobre seus conhecimentos relacionados à linha de cuidado do HIV e ao preparo profissional para atuar no atendimento de pessoas nesse contexto. Após a formação, todos os participantes passaram a considerar seus conhecimentos sobre PrEP, PEP e TARV como “muito bom/bom”, correspondendo a 19 estudantes (100%) em cada uma dessas dimensões, com diferença estatisticamente significativa em relação ao momento pré-formação (p < 0,001). Além disso, observou-se melhora significativa na percepção de preparo para acolher e orientar pessoas sobre a linha de cuidado do HIV no SUS, uma vez que 18 participantes (94,7%) referiram sentir-se “totalmente preparados/preparados” após a formação, em comparação a 10 (52,6%) antes da intervenção, com significância estatística pelo teste de Wilcoxon (p = 0,020).

Dessa forma, esses achados podem ser relacionados à problematização promovida pelas discussões dos casos clínicos, que possibilitaram a articulação entre ação e reflexão, favorecendo uma internalização crítica do conteúdo, e não apenas sua memorização^(11,13)^. Ao compartilharem dúvidas, experiências e interpretações durante a análise coletiva dos casos clínicos, os estudantes não apenas compreenderam os conteúdos trabalhados, mas também passaram a reconhecer suas próprias possibilidades de intervenção no cuidado, fortalecendo o compromisso ético-político com uma prática mais humana, integral e socialmente comprometida.

Estudo realizado com 63 enfermeiros também utilizou metodologia semelhante para a aplicação de uma formação, por meio de discussões em grupo e construção coletiva do conhecimento. Os achados aproximam-se dos resultados aqui apresentados, uma vez que os participantes relataram que a formação não se restringiu aos conceitos técnicos, mas também buscou estimular a consciência crítica sobre os determinantes sociais e culturais da saúde^(25)^.

Desse modo, os resultados encontrados podem servir como disparadores para o aprimoramento das estratégias curriculares nos cursos de enfermagem. Ao evidenciarem que a análise dialogada de casos clínicos fortalece a autonomia, a criticidade e a segurança dos estudantes, os achados apontam para a necessidade de ampliar práticas pedagógicas que aproximem teoria e realidade do cuidado. O enfoque ético nas simulações clínicas e nos estudos de caso favorece a tomada de decisão acolhedora e crítica, especialmente em contextos marcados pelo estigma, como o cuidado às pessoas que vivem com HIV^(24)^.

Por fim, destaca-se que formar profissionais críticos e comprometidos com o fortalecimento do SUS constitui parte essencial do papel social das universidades comunitárias. Em Santa Catarina, essas instituições representam uma realidade significativa e historicamente relevante na formação de trabalhadores da saúde. Ao assumirem esse compromisso, fortalecem não apenas a qualidade do ensino, mas também a construção de práticas de cuidado mais humanas, integrais e socialmente responsáveis.

Este estudo apresenta algumas limitações que devem ser consideradas na interpretação dos resultados. Embora a formação tenha sido direcionada a estudantes da 5ª à 10ª fase, participaram apenas acadêmicos da 8ª à 10ª fase. Esse fato esteve relacionado à organização curricular das demais fases, que possuíam aulas no mesmo horário em que a formação foi realizada, o que impossibilitou sua participação.

A pesquisa foi desenvolvida com três turmas de uma única universidade, o que pode limitar a generalização dos achados para outras instituições, regiões ou contextos. Embora a amostra tenha sido adequada aos objetivos propostos, seu tamanho foi relativamente pequeno. Além disso, destaca-se que os instrumentos utilizados foram baseados em autoavaliação, refletindo as percepções dos participantes acerca de seus conhecimentos e preparo para atuar na linha de cuidado do HIV, não constituindo uma medida objetiva de desempenho ou aquisição de conhecimento. Do mesmo modo, os resultados qualitativos derivam das experiências relatadas pelos participantes e da interpretação realizada pelos pesquisadores, estando sujeitos às particularidades do contexto investigado e às características inerentes às pesquisas de natureza qualitativa.

Apesar dessas limitações, este estudo evidencia a potência de uma formação construída de forma participativa e dialógica. A experiência demonstrou que, mesmo com um grupo reduzido, foi possível ampliar conhecimentos, fortalecer a segurança dos estudantes e aproximá-los da realidade do cuidado às pessoas que vivem com HIV. A articulação entre teoria e prática, por meio de casos clínicos contextualizados, favoreceu uma aprendizagem significativa e alinhada aos princípios do SUS, indicando que metodologias ativas podem gerar impactos relevantes no processo formativo e ser adaptadas a diferentes contextos de ensino em saúde.

## CONCLUSÃO

A formação em saúde participativa e dialógica desenvolvida com estudantes de enfermagem de uma universidade comunitária de Criciúma-SC mostrou-se efetiva para ampliar conhecimentos sobre o HIV e sua linha de cuidado, bem como para fortalecer a segurança dos participantes na abordagem às pessoas vivendo com HIV.

Os achados evidenciam que as lacunas identificadas não se limitam ao domínio técnico sobre PrEP, PEP e TARV, mas também envolvem dimensões éticas, sociais e subjetivas do cuidado. Nesse sentido, a experiência formativa reforça a importância de inserir, de forma mais intencional, metodologias ativas e espaços de diálogo na formação em enfermagem, favorecendo o desenvolvimento de profissionais mais críticos, sensíveis ao estigma e comprometidos com a integralidade do cuidado no SUS.

Assim, este estudo contribui ao propor uma experiência educativa que desmistifica não apenas o HIV, mas também a forma como essa temática é ensinada, valorizando práticas pedagógicas mais humanas e alinhadas ao cuidado integral em saúde.

## DISPONIBILIDADE DE DADOS

Todo o conjunto de dados que dá suporte aos resultados deste estudo foi publicado no próprio artigo.

# Translation: Desmitificando el VIH: capacitación académica para estudiantes de pregrado en enfermería

**DOI:** 10.1590/1980-220X-REEUSP-2026-0023es

**Authors:** Naomi Tamima Estevam Cipriano, Jacks Soratto, Cristiane Damiani Tomasi

**Affiliations:** 1 Universidade Federal de Santa Catarina, Florianópolis, SC, Brasil.; 2 Universidade do Extremo Sul Catarinense, Criciúma, SC, Brasil.

**Keywords:** VIH, Atención de Enfermería, Educación en Enfermería, Aprendizaje Basado en Problemas

## Abstract

**Objetivo:**

Desarrollar y evaluar una capacitación en salud, de carácter participativo y dialógico, con estudiantes del programa de enfermería de una universidad comunitaria de Criciúma/SC, sobre el Virus de Inmunodeficiencia Humana y su atención médica.

**Método:**

Investigación-acción, basada en la Pedagogía Crítica, realizada con 19 estudiantes de enfermería entre la 8.ª y la 10.ª fase del programa. La formación se llevó a cabo en cuatro etapas: exploratoria, planificación, acción y evaluación, con cuestionarios antes y después de la intervención.

**Resultados:**

Antes de la capacitación, predominaban las autoevaluaciones regulares o malas/muy malas sobre la profilaxis previa a la exposición, la profilaxis posterior a la exposición y la terapia antirretroviral. Tras la intervención, todos calificaron sus conocimientos como muy buenos o buenos. También se observó un aumento en la percepción de preparación para atender y orientar a las personas sobre la atención del Virus de Inmunodeficiencia Humana en el Sistema Único de Salud, del 52,6 % al 94,7 %.

**Conclusión:**

La formación en salud participativa y dialógica desarrollada con estudiantes de enfermería demostró ser eficaz para ampliar los conocimientos sobre el VIH y su atención, así como para fortalecer la seguridad de los participantes al abordar a las personas que viven con el VIH.

## INTRODUCCIÓN

El Virus de Inmunodeficiencia Humana (VIH) representa un importante problema de salud pública, ya que afecta a una parte significativa de la población y conlleva consecuencias clínicas, sociales y económicas relevantes, lo que agrava otros problemas de salud y aumenta los desafíos para los sistemas de atención y prevención^(1)^. En Brasil, entre 2007 y junio de 2023, se notificaron 489 594 nuevos casos de infección por el VIH, y al comparar los años 2020 y 2022, el número de casos de infección por el virus aumentó un 17,2 % en el país^(2)^.

El personal de enfermería desempeña un papel central en la atención integral del VIH/SIDA, desde la prevención hasta el seguimiento a largo plazo de las personas atendidas.

A este profesional le corresponde facilitar el acceso y la adherencia a las profilaxis pre y postexposición al VIH (PrEP y PEP), ofreciendo estas tecnologías de acuerdo con los criterios establecidos, además de desarrollar acciones de educación en salud, realizar pruebas rápidas de diagnóstico y dar seguimiento a los aspectos clínicos y psicosociales relacionados con el VIH y con las personas que ya reciben terapia antirretroviral (TARV)^(3)^.

A pesar de la importancia del papel del enfermero en esta línea de cuidados, los estudios señalan deficiencias en los conocimientos de estos profesionales sobre el VIH^(4,5,6,7)^. Esta realidad puede estar relacionada con un enfoque aún insuficiente del tema durante la formación en salud, lo que repercute directamente en la calidad de la atención y puede generar consecuencias individuales y colectivas, como la pérdida del plazo para el uso de la PEP y el aumento del riesgo de nuevas infecciones^(8,9)^.

De este modo, la mejora de la formación en salud, entendida como un proceso educativo, político y social que prepara a los profesionales mediante diferentes estrategias de enseñanza y aprendizaje^(10)^, constituye, especialmente en enfermería, un elemento importante para fortalecer las políticas públicas dirigidas a las personas que viven con el VIH. Al ampliar los conocimientos técnicos y promover una comprensión más sensible de las dimensiones sociales, éticas y subjetivas de la atención, la formación contribuye a una práctica profesional más equitativa, lo que acerca a los usuarios a la red de atención y mejora la calidad del acompañamiento ofrecido.

Considerando la importancia del papel del enfermero en la prevención y el tratamiento del VIH/SIDA y la brecha existente en el proceso de formación de los enfermeros, el presente estudio tuvo como objetivo desarrollar y evaluar un programa de capacitación en salud, de carácter participativo y dialógico, con estudiantes del curso de enfermería de una universidad comunitaria de la ciudad de Criciúma/SC, sobre el Virus de la Inmunodeficiencia Humana y su línea de cuidados.

## MÉTODO

### Tipo de Estudio

Se utilizó la investigación-acción como metodología de este estudio, la cual enfoca sus esfuerzos en la propuesta y aplicación de una intervención que modifique la realidad presentada por el investigador y los participantes del contexto observado^(11)^. Para Thiollent, la investigación-acción implica, en general, la identificación de un problema colectivo, la planificación de una acción, la participación de los sujetos involucrados, la implementación de la intervención, la evaluación de los resultados y la producción conjunta de conocimiento^(12)^.

Además, se utilizó un marco teórico basado en los principios de la Pedagogía Crítica descritos por Paulo Freire, cuyo núcleo se presenta como las manifestaciones de las relaciones de poder y desigualdad, cuestionándolas en los espacios educativos^(13)^. El conocimiento se construye de manera conjunta, en la que ambas partes ocupan las posiciones de educador y educando, a través del diálogo y el intercambio de conocimientos^(13)^.

El informe de los aspectos cualitativos del estudio se orientó por las recomendaciones de los Criterios Consolidados para el Informe de Investigación Cualitativa (COREQ), traducidos y validados al idioma portugués^(14)^. El diseño y la recolección de datos estuvieron a cargo de la primera autora, enfermera especialista en salud colectiva y estudiante de maestría en Enfermería, con experiencia previa en educación en salud y VIH y práctica en atención primaria de salud a través de la residencia multiprofesional en salud colectiva. La investigadora no tenía ninguna relación jerárquica ni evaluativa con los participantes, aunque compartía con ellos el entorno institucional universitario. Durante el desarrollo del estudio, se buscó minimizar posibles sesgos mediante el uso de instrumentos estandarizados para la recolección de datos, el registro sistemático de las etapas de la investigación y el análisis articulado entre los datos cuantitativos y cualitativos, respetando los supuestos de la investigación-acción y la pedagogía crítica.

### Lugar, Población, Criterios de Selección y Definición de la Muestra

El estudio se llevó a cabo en una universidad comunitaria de la ciudad de Criciúma, en el estado de Santa Catarina, Brasil. Los criterios de inclusión fueron los estudiantes matriculados en la carrera de enfermería entre la 5.ª y la 10.ª fase del curso en dicha universidad, mayores de 18 años; y los criterios de exclusión fueron los estudiantes que declararon no tener tiempo disponible para participar en la capacitación.

La población elegible estaba compuesta por 250 estudiantes, y se les invitó a todos a participar. De ellos, 40 manifestaron interés en participar en el estudio. Sin embargo, la muestra final estuvo compuesta por 19 participantes, ya que los demás no tenían disponibilidad para participar en las etapas previstas en la investigación, lo que caracterizó un muestreo por conveniencia. Esta limitación se debió a la organización de las clases en turnos diurnos y nocturnos, así como al perfil del programa, caracterizado por un número significativo de estudiantes que compaginan la formación académica con actividades laborales.

### Protocolo del Estudio

El estudio se organizó en cuatro etapas: exploratoria, de planificación, de acción y de evaluación^(11)^, las cuales se resumen en el Cuadro 1.

**Cuadro 1:** Etapas de la construcción y el desarrollo de la formación en salud sobre el VIH para estudiantes de enfermeira – Criciúma, SC, Brasil, 2025.

Etapa	Objetivo	Participación de los estudiantes	Estrategias utilizadas	Fundamentación freireana	Producto generado
*Exploratoria*	Identificar las necesidades de capacitación sobre el VIH	Respondieron un cuestionario de diagnóstico autoadministrado	Cuestionario de Google Forms	Investigación temática, con el objetivo de comprender la realidad y las necesidades de los participantes	Análisis de las brechas en materia de PrEP, PEP, TARV y atención de acogida
*Planificación*	Estructurar la capacitación	Las inquietudes de los estudiantes sirvieron de base para la planificación	Definición de competencias, objetivos y casos clínicos	Análisis crítico de la realidad, transformando las necesidades identificadas en temas generadores para la formación	Plan de capacitación
*Acción*	Ampliar los conocimientos sobre la atención del VIH	Participación activa en grupos	Exposición interactiva, discusión de casos clínicos, elaboración de mapas mentales	Diálogo, construcción compartida del conocimiento y praxis	Construcción colectiva del conocimiento
*Evaluación*	Evaluar el aprendizaje y la eficacia de la capacitación	Autoevaluación y evaluación de la actividad	Cuestionario autoadministrado	Reflexión crítica sobre la práctica y reconstrucción del conocimiento	Identificación de fortalezas y aspectos a mejorar

Fuente: Elaborado por los autores, 2026.

La organización de la formación en salud se basó en los supuestos de la pedagogía crítica freireana^(13)^, especialmente en la investigación temática, la problematización de la realidad, la dialogicidad y la praxis. Estos supuestos se incorporaron en todas las etapas de la investigación-acción, desde la identificación de las necesidades de los estudiantes hasta la evaluación crítica de la intervención realizada.

En la fase exploratoria, la primera autora realizó la invitación en persona en las aulas de los grupos seleccionados, presentando la propuesta de investigación e invitando a los estudiantes a participar. A continuación, se puso a disposición un cuestionario autoadministrado elaborado en la plataforma *Google Forms*, al que se accedía mediante un código *QR*, con preguntas enfocadas en la autopercepción de los participantes sobre su comprensión de la atención del VIH, incluyendo la PrEP, la PEP y el TARV, así como las principales dificultades experimentadas en relación con el tema.

Las respuestas obtenidas a través del formulario constituyeron el diagnóstico inicial de la intervención educativa y permitieron identificar las principales necesidades de formación señaladas por los estudiantes. Entre ellas, se destacaron dudas relacionadas con la actuación del personal de enfermería en la atención del VIH, el manejo y la orientación sobre la PrEP, la PEP y el TARV, la prestación de una atención integral y humanizada, además de inseguridades respecto al enfoque clínico y educativo ante situaciones prácticas que involucran a personas que viven con el VIH o en riesgo de infección.

De esta manera, el problema se definió conjuntamente con los participantes, con el objetivo de garantizar que el tema se ajustara a sus necesidades y percepciones, además de fomentar reflexiones capaces de impulsar las transformaciones deseadas. A partir de este diagnóstico inicial, se inició la etapa de planificación, en la que se estableció la competencia que se debía alcanzar tras la aplicación de la capacitación: comprender la actuación del personal de enfermería en la atención del VIH de manera integral y holística. Con base en las respuestas del formulario, se definieron los contenidos prioritarios de la capacitación y se optó por la resolución de cuatro casos clínicos, que abarcaban situaciones relacionadas con la prevención, el seguimiento, el tratamiento y el enfoque integral en la atención del VIH^(15)^.

Se optó por el uso de estudios de caso, ya que estos se presentan como una estrategia pedagógica que fortalece el proceso de formación en salud, dado que el análisis de casos es una estrategia de enseñanza dinámica e interactiva que contribuye a mejorar las habilidades de pensamiento crítico y resolución de problemas^(16)^. En este formato, los estudiantes experimentan la praxis freiriana, entendida como el movimiento continuo entre acción, reflexión y nueva acción, en el que el pensamiento crítico sobre la realidad sirve de fundamento para transformarla^(11)^.

La combinación de exposición dialogada, discusión de casos clínicos, construcción de mapas mentales y trabajo en grupos pequeños se definió por su consonancia con los supuestos de la investigación-acción y la pedagogía crítica freiriana, lo que favoreció la problematización de situaciones concretas, el intercambio de experiencias entre los participantes, la construcción colectiva del conocimiento y el desarrollo de la reflexión crítica sobre la práctica de la enfermería en la atención del VIH.

En la fase de acción, se llevó a cabo la aplicación de la capacitación académica en una fecha previamente acordada y programada entre la investigadora y los estudiantes, en un salón cedido por la universidad, con una duración de 4 horas. La sesión comenzó con una breve explicación de la investigadora sobre la diferencia entre el VIH y el SIDA, los comportamientos de riesgo y las poblaciones clave, la epidemiología de la infección y el mandala de la prevención combinada.

Posteriormente, los participantes se dividieron en cuatro grupos, a cada uno de los cuales se le proporcionó un caso clínico con preguntas orientativas, el mandala de la prevención combinada, la ficha de notificación de VIH/SIDA y los formularios de prescripción de la PEP, la PrEP y el TARV; así como cartulina y bolígrafos para elaborar un mapa mental.

Los casos clínicos revelaron realidades distintas que exigen del personal de enfermería sensibilidad y una postura ética. En el primero, un joven buscó ayuda tras una relación sexual sin protección, lo que abrió un espacio para hablar sobre los criterios de la PEP y la importancia de una atención sin juicios. En el segundo, una trabajadora sexual acudió a la Unidad Básica de Salud para hacerse la prueba, lo que puso de manifiesto la necesidad de garantizar la confidencialidad, el respeto y la información clara sobre la PEP y la PrEP. El tercer caso presentó la historia de un hombre viviendo en la calle y consumidor de drogas inyectables, que vivía con el VIH sin tratamiento y presentaba síntomas indicativos de una infección oportunista, lo que llevó a reflexionar sobre el diagnóstico y sobre cómo apoyar su adherencia al tratamiento. Por último, el cuarto caso presentó a una mujer embarazada que descubrió su diagnóstico de VIH durante la atención prenatal, una situación que requirió orientación, apoyo emocional, derivaciones adecuadas y un enfoque cuidadoso con respecto a su pareja.

Los casos clínicos fueron elaborados por las investigadoras y se basaron en los datos epidemiológicos del VIH en Brasil, en los protocolos vigentes del Ministerio de Salud y en la práctica de la enfermería en la atención a las personas que viven con el VIH.

En esta etapa, el conocimiento se construyó de manera colectiva, a partir de las experiencias, percepciones y conocimientos previos de los estudiantes en la resolución de los casos clínicos. La investigadora actuó como mediadora de los intercambios, dirigiendo y profundizando en las discusiones con el fin de fomentar la reflexión crítica sobre el tema. De esta manera, se incorporaron los principios de la investigación-acción y la pedagogía crítica, especialmente en lo que se refiere a la participación activa de los estudiantes en el proceso de aprendizaje.

Ese mismo día se llevó a cabo la última etapa de la investigación, destinada no solo a evaluar los conocimientos desarrollados por los estudiantes, sino también a analizar la propia formación académica y su efectividad. Para ello, se aplicó un cuestionario autoadministrado, elaborado para identificar los efectos de la acción educativa y evaluar la actividad implementada.

El instrumento constaba de tres dimensiones: organización y didáctica de la formación; aprendizaje y desarrollo; y aplicabilidad e impacto. Esta etapa permitió identificar potencialidades y aspectos que deben mejorarse, lo que facilitó ajustes en la formación antes de su aplicación en otros contextos o instituciones.

La dimensión “organización y didáctica de la capacitación” del cuestionario constaba de tres afirmaciones: *el contenido de la capacitación se presentó de manera clara y comprensible; la metodología utilizada fue adecuada para los objetivos de la capacitación; y los materiales didácticos (presentaciones, textos, videos, etc.) fueron de buena calidad*.

Por su parte, la dimensión “aprendizaje y desarrollo” constaba de cuatro preguntas*: ¿cómo calificarías tu conocimiento sobre las indicaciones, los comportamientos de riesgo, la duración del tratamiento y los efectos secundarios de la profilaxis preexposición (PrEP)?; ¿cómo calificarías tu conocimiento sobre las indicaciones, los comportamientos de riesgo, la duración del tratamiento y los efectos secundarios de la profilaxis postexposición (PEP)?; ¿cómo calificarías tu conocimiento sobre las indicaciones, los comportamientos de riesgo, la duración del tratamiento y los efectos secundarios de la terapia antirretroviral (TARV)?;* y *¿te sientes preparado(a) para atender y orientar a las personas en el Sistema Único de Salud sobre la atención del VIH?*.

Las preguntas relacionadas con la autopercepción del conocimiento y la preparación para actuar en la atención del VIH se evaluaron mediante una escala de Likert de cinco puntos, que variaba de 1 (muy malo o totalmente desprevenido) a 5 (muy bueno o totalmente preparado). A efectos de la presentación de los resultados, las respuestas se agruparon en tres categorías: muy bueno/bueno, regular y malo/muy malo para las preguntas relacionadas con el conocimiento; y totalmente preparado/preparado, neutro e imprepado/totalmente imprepado para la pregunta referente a la preparación para atender y orientar a las personas en la atención del VIH.

Por último, la dimensión “aplicabilidad e impacto” se componía de dos afirmaciones: “el contenido de la capacitación es relevante para mi práctica académica y mi futura actividad profesional”; y “el tema abordado debería tener mayor presencia en el plan de estudios de la carrera de enfermería”.

Tras la finalización de la capacitación académica, se puso a disposición de los estudiantes un material didáctico que contenía un breve resumen de los puntos principales abordados. Este material tenía como objetivo servir de apoyo para su consulta durante las pasantías y las prácticas clínicas, facilitando la revisión de los contenidos trabajados en la capacitación.

Además, el proceso se sistematizó y dio lugar a la elaboración de un material educativo titulado “Guía de capacitación sobre la actuación del enfermero en la atención del VIH/SIDA”, elaborado por los autores. El material presenta paso a paso la capacitación en salud desarrollada en el estudio, incluyendo su organización, los contenidos abordados y las estrategias utilizadas, con el fin de permitir su replicación en otras universidades e instituciones de salud. De esta manera, busca servir como apoyo teórico-práctico para la formación y capacitación de estudiantes y profesionales de enfermería en el abordaje de la temática del VIH/SIDA.

### Análisis y Procesamiento de los Datos

Los datos cuantitativos recopilados en los cuestionarios aplicados antes y después de la capacitación se organizaron inicialmente en una tabla de Excel.

Los análisis estadísticos se realizaron utilizando el *software IBM SPSS Statistics*. Las variables se presentaron en frecuencias absolutas y relativas. Para comparar las evaluaciones antes y después de la capacitación, se utilizó el test de Wilcoxon^(17)^ para muestras emparejadas, considerando la distribución no paramétrica de los datos. El nivel de significancia adoptado fue del 5 % (p < 0,05).

Además, los datos cualitativos, procedentes de las preguntas abiertas, se sometieron al análisis de contenido propuesto por Bardin^(18)^, siguiendo las etapas de preanálisis, exploración del material e interpretación de los resultados. Inicialmente, se realizó una lectura exhaustiva de las respuestas, seguida de la identificación de unidades de sentido relacionadas con las percepciones de los estudiantes sobre la capacitación y sobre la enseñanza del VIH en la carrera universitaria. Posteriormente, los contenidos se agruparon por similitud temática, lo que dio lugar a las categorías analíticas presentadas en los resultados. Para garantizar el anonimato de los participantes, los fragmentos de las declaraciones se identificaron con la letra “e” seguida de una numeración secuencial (e1, e2, e3...), correspondiente al orden de participación en el estudio.

### Aspectos Éticos

El proyecto de este estudio fue presentado y aprobado por el Comité de Ética en Investigación de la institución, bajo el dictamen n.º 7.606.538 y el CAAE n.º 88937925.6.0000.0119. Los estudiantes que aceptaron participar en la investigación completaron y firmaron el Formulario de Consentimiento Libre e Informado antes del inicio de los procedimientos de investigación. Para garantizar el anonimato, cada participante fue identificado mediante un código alfanumérico, designándose como e1, e2, e3 y así sucesivamente.

## RESULTADOS

Los resultados se presentan en dos etapas: la de desarrollo de la formación en salud y la de evaluación de la formación en salud.

La fase de desarrollo de la formación en salud se construyó a partir de las necesidades identificadas por los estudiantes en la etapa exploratoria de la investigación-acción. Los datos recopilados evidenciaron brechas relacionadas con el conocimiento sobre la PrEP, la PEP, el TARV y la actuación del personal de enfermería en la atención del VIH, además de la percepción de un enfoque superficial del tema durante la carrera.

Con base en este diagnóstico, se definió como competencia central de la formación comprender la actuación de la enfermería en la atención del VIH de manera integral y holística. Para alcanzar esta competencia, se establecieron objetivos de aprendizaje enfocados en la prevención combinada, el diagnóstico, el tratamiento y la atención de las personas que viven con el VIH.

La capacitación se organizó en una sesión presencial de cuatro horas de duración, estructurada a partir de una exposición dialogada, la discusión de casos clínicos y la construcción colectiva de mapas mentales. Se elaboraron cuatro casos clínicos que representaban diferentes contextos de atención relacionados con el VIH, abarcando situaciones que implicaban la indicación de la PEP, el uso de la PrEP, la adherencia al tratamiento antirretroviral y el seguimiento de mujeres embarazadas que viven con el VIH.

Como resultado de la investigación, se desarrolló el material educativo titulado “*Guía de capacitación sobre la actuación del personal de enfermería en la atención del VIH/SIDA*” (*Guia de formação sobre a atuação do enfermeiro na linha de cuidado do HIV/AIDS,* en portugués*)*, que contiene la sistematización de la propuesta educativa, los contenidos trabajados y las estrategias pedagógicas utilizadas, con potencial para ser replicado en instituciones educativas y servicios de salud^(19)^.

La muestra estuvo compuesta por 19 estudiantes, todos ellos entre el octavo y el décimo semestre de la carrera de enfermería. Se incluyeron 17 mujeres cisgénero y 2 hombres cisgénero; 12 tenían entre 20 y 30 años, 5 entre 31 y 40 años y 2 entre 41 y 50 años; 14 se declararon solteros, 4 casados y 1 en unión estable.

La Tabla 1 presenta los resultados de la dimensión “aprendizaje y desarrollo”, antes y después de la aplicación de la capacitación. También en esta dimensión, los comentarios de los estudiantes pusieron de manifiesto que, aunque el tema ya se había abordado a lo largo del plan de estudios, dicho abordaje se realizó de manera limitada, sin suficiente profundidad. Estos relatos se organizaron en la categoría “Abordaje curricular superficial del tema”, representada por los siguientes comentarios: “Sí, de manera superficial” (e4); “No nos profundizamos” (e12); y “Sí, pero contenido básico” (e14).

**Tabla 1:** Autoevaluación de los estudiantes sobre sus conocimientos respecto a la atención del VIH, comparación entre antes y después de la formación académica, frecuencia absoluta y relativa – Criciúma, SC, Brasil, 2025. (n = 19).

Pregunta de evaluación	Cuestionario pre-capacitación n (%)	Cuestionario post-capacitación n (%)	p-valor (test de Wilcoxon)
Evaluación de los conocimientos sobre la PrEP			
Muy bueno/Bueno	4 (21,1%)	19 (100%)	p < 0,001
Regular	13 (68,4%)	0 (0%)	
Mal/Muy mal	2 (10,5%)	0 (0%)	
Evaluación de los conocimientos sobre la PEP			
Muy bueno/Bueno	3 (15,8%)	19 (100%)	p < 0,001
Regular	13 (68,4%)	0 (0%)	
Mal/Muy mal	3 (15,8%)	0 (0%)	
Evaluación de los conocimientos sobre el TARV			
Muy bueno/Bueno	2 (10,5%)	19 (100%)	p < 0,001
Regular	12 (63,2%)	0 (0%)	
Mal/Muy mal	5 (26,3%)	0 (0%)	
Evaluación de la preparación para atender y orientar a las personas sobre la cadena de atención del VIH en el SUS			
Totalmente preparado/Preparado	10 (52,6%)	18 (94,7%)	p = 0,020
Neutro	8 (42,1%)	1 (5,3%)	
Despreparado/Totalmente despreparado	1 (5,3%)	0 (0%)	

Fuente: Elaborado por los autores, 2026.

La Figura 1 presenta gráficamente los resultados de las dimensiones “organización y didáctica de la capacitación” y “aplicabilidad e impacto”, recogidos tras la capacitación.

**Figura 1:** Evaluación de la capacitación en cuanto a organización, didáctica, aplicabilidad e impacto – Criciúma, SC, Brasil, 2025. (n = 19).

Fuente: Elaborado por los autores, 2026.

Por último, la categoría “Potencialidades de la formación en salud” reúne los comentarios de los estudiantes sobre los aspectos positivos de la actividad. Los relatos indican que la formación se destacó no solo por el contenido abordado, sino también por la forma en que se llevó a cabo, con énfasis en la claridad de las explicaciones, la calidad de la didáctica, la dinámica propuesta y la participación activa de los estudiantes. Esta percepción se refleja en los comentarios: “La didáctica y los métodos utilizados” (e3); “Me gustó mucho la dinámica y la forma en que se abordaron los temas, me cautivó bastante” (e4); “La didáctica presentada, la discusión y el estudio de caso” (e11); “Lo que más me gustó de la capacitación fue la forma clara y actualizada de transmitir conocimientos que serán útiles en la práctica profesional” (e14); y “Además de todo, la participación de cada estudiante” (e15).

## DISCUSIÓN

Los participantes de la presente investigación evaluaron, antes de la implementación de la capacitación, sus conocimientos sobre la PEP, la PrEP y el TARV, en su mayoría, como regulares. Se observó un resultado similar en una investigación realizada con estudiantes de enfermería de una universidad en la India, que evaluó los conocimientos sobre el VIH/SIDA, el tratamiento y las profilaxis, identificando niveles regulares o bajos de comprensión sobre los criterios de indicación y el tiempo de uso de la PEP, así como sobre las formas de transmisión de la infección^(8)^. En el contexto nacional, los estudios también evidencian brechas en el conocimiento sobre profilaxis y cuidados dirigidos a esta población específica^(6,20,21)^.

En este sentido, una investigación realizada con enfermeros de la capital de Santa Catarina señala el escaso tratamiento del tema durante la carrera como uno de los principales factores relacionados con la insuficiencia de conocimientos^(22)^. Mientras que los profesionales que ya se encuentran en el mercado laboral informan no haber recibido la formación adecuada durante su formación inicial, los estudiantes participantes en este estudio confirman que, aunque el tema se aborda en el aula, esto ocurre de manera superficial y rápida, con mayor énfasis en los aspectos biológicos de la infección, sin profundizar en el tratamiento y la atención integral que se debe brindar a las personas que viven con el VIH.

Este panorama pone de manifiesto una brecha formativa que puede afectar directamente la práctica clínica de los profesionales y académicos, ya que los participantes manifestaron sentimientos de falta de preparación e inseguridad para atender a personas que se encuentran en la cadena de atención del VIH. Otro estudio también señala esta realidad y refuerza que, para que los futuros profesionales puedan orientar con seguridad, acogida y responsabilidad, es necesario que la formación ofrezca oportunidades concretas de aprendizaje sobre el tema^(23)^.

Tanto la investigación-acción como la pedagogía crítica y dialógica propuesta por Paulo Freire se presentan como alternativas poderosas para el trabajo en la atención del VIH, ya que favorecen la construcción del conocimiento de manera conjunta, horizontal y contextualizada, a partir de la problematización de situaciones reales y del diálogo.

En este sentido, ambas se alinean con el concepto de educación liberadora, al romper con el modelo bancario de enseñanza y favorecer la formación de sujetos críticos, autónomos y capaces de intervenir en la realidad^(11)^.

La aplicación del método freiriano en esta formación permitió que los estudiantes asumieran un papel activo en el proceso de aprendizaje, dejando atrás la posición pasiva de receptores de contenido para reconocerse como sujetos críticos, reflexivos y corresponsables de la construcción del conocimiento. El diálogo establecido entre la investigadora y los estudiantes favoreció el intercambio de experiencias y conocimientos, valorando los conocimientos previos de los participantes y promoviendo una reflexión crítica sobre el papel del enfermero frente a las desigualdades sociales y la estigmatización asociada al VIH.

En este sentido, la discusión se amplió a la formación ética y política del enfermero, desplazando el enfoque exclusivamente de la enfermedad hacia la persona que vive con el VIH, tomando en cuenta su historia, su contexto y sus derechos. De este modo, la formación se configuró también como un espacio de concientización y empoderamiento, contribuyendo a la repolitización de la formación en salud, un aspecto esencial para la defensa de la atención integral y para hacer frente al estigma relacionado con el VIH. Al fortalecer el compromiso ético-político de estos futuros profesionales con la vida y los derechos humanos, la experiencia reafirma el papel de la educación liberadora en la construcción de prácticas de atención más humanas, críticas y socialmente comprometidas^(24)^.

La capacitación demostró ser eficaz y bien aceptada por los estudiantes, tal como lo evidencian los resultados de la evaluación, en la que el 100 % de los participantes calificaron la metodología utilizada como adecuada para los objetivos propuestos. Además, algunos estudiantes destacaron la didáctica como uno de los puntos fuertes de la capacitación, ya que favoreció la participación del grupo y el intercambio de conocimientos. Desde el punto de vista de la investigadora, el compromiso de los participantes también fue fundamental para el buen desarrollo de la actividad.

Además del compromiso de los estudiantes, la capacitación presentó resultados importantes en cuanto a la autopercepción de los participantes sobre sus conocimientos relacionados con la atención del VIH y la preparación profesional para atender a personas en este contexto. Tras la capacitación, todos los participantes pasaron a considerar sus conocimientos sobre la PrEP, la PEP y el TARV como “muy buenos/buenos”, lo que correspondió a 19 estudiantes (100 %) en cada una de estas dimensiones, con una diferencia estadísticamente significativa en comparación con el momento previo a la capacitación (p < 0,001). Además, se observó una mejora significativa en la percepción de preparación para recibir y orientar a las personas sobre la atención del VIH en el SUS, ya que 18 participantes (94,7 %) indicaron sentirse “totalmente preparados/preparados” después de la capacitación, en comparación con 10 (52,6 %) antes de la intervención, con significación estadística según el test de Wilcoxon (p = 0,020).

De este modo, estos hallazgos pueden relacionarse con la reflexión promovida por las discusiones de los casos clínicos, que permitieron la articulación entre acción y reflexión, favoreciendo una interiorización crítica del contenido, y no solo su memorización^(11,13)^. Al compartir dudas, experiencias e interpretaciones durante el análisis colectivo de los casos clínicos, los estudiantes no solo comprendieron los contenidos trabajados, sino que también comenzaron a reconocer sus propias posibilidades de intervención en la atención, fortaleciendo el compromiso ético-político con una práctica más humana, integral y socialmente comprometida.

Un estudio realizado con 63 enfermeros también utilizó una metodología similar para la implementación de una capacitación, a través de discusiones grupales y la construcción colectiva del conocimiento. Los hallazgos se asemejan a los resultados aquí presentados, ya que los participantes informaron que la capacitación no se limitó a los conceptos técnicos, sino que también buscó estimular la conciencia crítica sobre los determinantes sociales y culturales de la salud^(25)^.

De este modo, los resultados obtenidos pueden servir como punto de partida para mejorar las estrategias curriculares en los programas de enfermería. Al evidenciar que el análisis dialogado de casos clínicos fortalece la autonomía, el espíritu crítico y la seguridad de los estudiantes, los hallazgos apuntan a la necesidad de ampliar las prácticas pedagógicas que acerquen la teoría y la realidad de la atención. El enfoque ético en las simulaciones clínicas y en los estudios de caso favorece la toma de decisiones inclusiva y crítica, especialmente en contextos marcados por el estigma, como la atención a las personas que viven con el VIH^(24)^.

Por último, cabe destacar que formar profesionales críticos y comprometidos con el fortalecimiento del SUS constituye una parte esencial del papel social de las universidades comunitarias. En Santa Catarina, estas instituciones representan una realidad significativa e históricamente relevante en la formación de trabajadores de la salud. Al asumir este compromiso, fortalecen no solo la calidad de la enseñanza, sino también la construcción de prácticas de atención más humanas, integrales y socialmente responsables.

Este estudio presenta algunas limitaciones que deben tenerse en cuenta al interpretar los resultados. Aunque la capacitación se dirigió a estudiantes de la 5.ª a la 10.ª fase, solo participaron estudiantes de la 8.ª a la 10.ª fase. Esto se debió a la organización curricular de las demás fases, que tenían clases en el mismo horario en que se llevó a cabo la capacitación, lo que impidió su participación.

La investigación se llevó a cabo con tres grupos de una sola universidad, lo que puede limitar la generalización de los hallazgos a otras instituciones, regiones o contextos. Aunque la muestra fue adecuada para los objetivos propuestos, su tamaño fue relativamente pequeño. Además, cabe destacar que los instrumentos utilizados se basaron en la autoevaluación, lo que refleja las percepciones de los participantes sobre sus conocimientos y preparación para trabajar en la atención del VIH, sin constituir una medida objetiva del desempeño o la adquisición de conocimientos.

Del mismo modo, los resultados cualitativos se derivan de las experiencias relatadas por los participantes y de la interpretación realizada por los investigadores, por lo que están sujetos a las particularidades del contexto investigado y a las características inherentes a las investigaciones de naturaleza cualitativa.

A pesar de estas limitaciones, este estudio pone de manifiesto el potencial de una formación construida de manera participativa y dialógica. La experiencia demostró que, incluso con un grupo reducido, fue posible ampliar los conocimientos, fortalecer la seguridad de los estudiantes y acercarlos a la realidad de la atención a las personas que viven con el VIH. La articulación entre teoría y práctica, a través de casos clínicos contextualizados, favoreció un aprendizaje significativo y alineado con los principios del SUS, lo que indica que las metodologías activas pueden generar impactos relevantes en el proceso formativo y adaptarse a diferentes contextos de enseñanza en salud.

## CONCLUSIÓN

La formación en salud participativa y dialógica desarrollada con estudiantes de enfermería de una universidad comunitaria de Criciúma (SC) demostró ser eficaz para ampliar los conocimientos sobre el VIH y su atención, así como para fortalecer la seguridad de los participantes al abordar a las personas que viven con el VIH.

Los hallazgos evidencian que las brechas identificadas no se limitan al dominio técnico sobre la PrEP, la PEP y el TARV, sino que también involucran dimensiones éticas, sociales y subjetivas de la atención. En este sentido, la experiencia formativa refuerza la importancia de incorporar, de manera más intencional, metodologías activas y espacios de diálogo en la formación en enfermería, lo que favorece el desarrollo de profesionales más críticos, sensibles al estigma y comprometidos con la atención integral en el SUS.

Así, este estudio contribuye al proponer una experiencia educativa que desmitifica no solo el VIH, sino también la forma en que se enseña este tema, valorando prácticas pedagógicas más humanas y alineadas con la atención integral en salud.

## DISPONIBILIDAD DE DATOS

Todo el conjunto de datos que respalda los resultados de este estudio se publicó en el propio artículo.
